# Clinical Detection of Primary Pulmonary Angiosarcoma

**DOI:** 10.7759/cureus.17059

**Published:** 2021-08-10

**Authors:** Kaynat Khalid, Anas Khan, Christine M Lomiguen, Justin Chin

**Affiliations:** 1 Department of Primary Care, Lake Erie College of Osteopathic Medicine, Erie, USA; 2 Department of Medical Education, Lake Erie College of Osteopathic Medicine, Erie, USA; 3 Department of Family Medicine, Millcreek Community Hospital, Erie, USA; 4 Department of Family Medicine, LifeLong Medical Care, Richmond, USA

**Keywords:** pulmonary angiosarcoma, cancer, saddle pulmonary embolism, atrial fibrillation, syncope, pulmonary tumor, metastatic cancer, vascular tumor, primary angiosarcoma, pas

## Abstract

Pulmonary angiosarcomas (PAS) are rare malignant vascular tumors that due to their aggressive and metastatic nature, are often diagnosed at a late stage, resulting in a poorer prognosis. Here we present a 53-year-old male who was initially found to have recurring episodes of dyspnea and syncope, with initial workup showing bilateral saddle pulmonary embolisms on computerized tomography, presumed secondary to newly discovered atrial fibrillation with sinus node dysfunction. Further investigation over subsequent months and subsequent biopsy of a potential lung mass ultimately revealed pulmonary angiosarcoma of the spindle cell line. This case emphasizes findings in the current literature, which reveal the time between the onset of symptoms and definitive diagnosis ranges from two to six months, with a median survival time of seven months or two months, with solitary lesions or multiple lesions at the time of diagnosis, respectively. With the limited incidence of PAS, this case suggests benefits in the development of screening and detection criteria for earlier detection and treatment.

## Introduction

Angiosarcomas are malignant endothelial neoplasms that commonly affect the skin, soft tissue/fascia, breast, and liver. Due to their aggressive and metastatic nature, angiosarcomas are often diagnosed at a late stage, resulting in a poorer prognosis [1]. First described in 1923 after reclassification of a primary cardiac tumor, pulmonary angiosarcomas (PAS) are classically found on the pulmonary arteries [2,3]. Subsequent literature and studies have been sparse, as PAS is frequently misdiagnosed due to a lack of defining symptoms as well as symptoms that more closely resemble more common diseases or conditions, such as thromboembolism [4,5]. Nevertheless, early detection and diagnosis have been reported, allowing for prompt resection of the tumor and increased odds of remission [6,7]. Due to the lack of proper diagnostic criteria, PAS is often misdiagnosed or underdiagnosed, leading to progression of the disease and high mortality [8,9].

Here we present a case of PAS in a 53-year-old male with recurring episodes of dyspnea and syncope that resulted in bilateral pulmonary embolisms. Common presentations and literature of PAS are also reviewed. 

## Case presentation

A 53-year-old Middle Eastern male with no formal past medical or surgical history presented to the emergency room in New York following a syncopal episode while walking at work. Prior to this, he had never experienced syncopal episodes or any pre-syncopal symptoms. On initial workup, the patient had bilateral saddle embolisms on computerized tomography, presumed secondary to a newly discovered atrial fibrillation with sinus node dysfunction. The patient denied episodes of palpitations or exertional chest pain prior to the discovery of atrial fibrillation. Cardiology and pulmonology services advised pacemaker versus implantable cardioversion defibrillator placement with appropriate anticoagulation and thrombolytics. While awaiting treatment, a cardiothoracic service was consulted for a second opinion for the concern of thoracic malignancy as the primary cause.

The patient was transferred to a tertiary care center for further testing and biopsy over the next two months, which ultimately revealed pulmonary angiosarcoma of the spindle cell line. The patient was started on a course of ifosfamide and adriamycin and frequently returned for treatment and sequelae associated with pulmonary hypertension and symptom progression. Due to the large size, poor prognosis, and spread of his mass, he was not a candidate for resection, with eventual enlargement and localized spread. In the following six to eight months, the patient attempted naturopathic and complementary therapies, which included herbal, spiritual, and cultural remedies, in addition to his chemotherapy, with limited improvement in symptoms.

One year after his initial diagnosis, the patient passed away on palliative pain management after an episode of dyspnea and chest pain.

## Discussion

**Figure 1:**
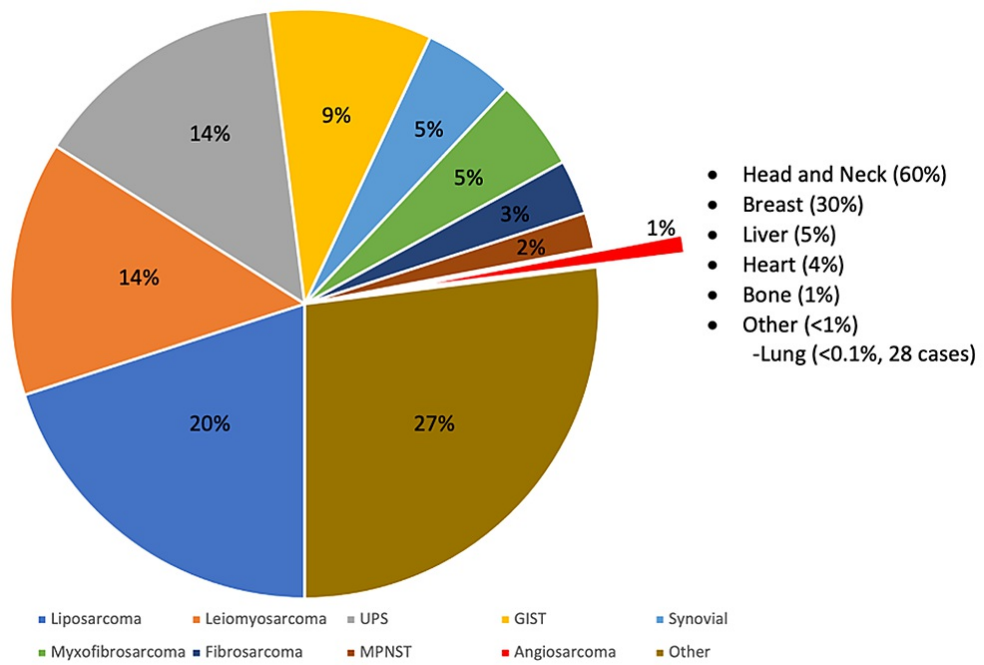
Relative distribution of soft tissue sarcomas in the adult population. Angiosarcomas comprise 1% of cases, with head/neck and breast being the most common. Primary pulmonary angiosarcomas make up <0.1% of cases. UPS = undifferentiated pleomorphic sarcoma; MPNST = malignant peripheral nerve sheath tumor; GIST = gastrointestinal stromal tumor. Adapted from the data published by Memorial Sloan Kettering Cancer Center [11].

Primary pulmonary angiosarcoma is an aggressive and rare form of pulmonary artery malignancy. Secondary pulmonary angiosarcoma, in which metastasis to the lung is from another primary site such as the heart or breast, is more common [10]. Similar to other angiosarcomas, PAS is derived from vascular endothelial cells [1,2]. To this date, less than 30 cases have been reported and described in the literature, resulting in an incidence rate of less than 0.030% of the general population (Figure 1) [11]. While cases of PAS have been found predominantly in middle-aged males, very little is known about its clinical course, prognosis, or other disease predisposition due to its rarity [12,13]. Currently, there are no established criteria to screen or triage cases that resemble PAS as its rarity precludes its inclusion in the differential diagnosis of common healthcare providers. 

As seen in previous reports of PAS, one of the largest obstacles to treatment is prompt detection. The average time between the presentation of initial symptoms to biopsy can range widely, from two to six months, resulting in delayed diagnosis, treatment, and overall care [8,14]. As seen in this case, although two months elapsed between initial hospitalization and biopsy, the patient had concerning symptoms for over a year. Misdiagnosis is common in PAS as symptoms are nonspecific and mimic other more common disease processes [15]. Nonspecific constitutional and respiratory symptoms can include weight loss, shortness of breath, cough, hemoptysis, and chest pain, for which the differential diagnoses encompass more common diseases in various organ systems. In the case presentation, the bilateral saddle embolisms were attributed to the new-onset atrial fibrillation rather than the hypercoagulable state of PAS. Exertional dyspnea and syncope are prevalent symptoms in many cardiovascular pathologies, including PAS. Due to lack of diagnostic criteria, recognition and subsequent treatment of PAS was delayed in this case, resulting in rapid disease progression that precluded the possibility of remission or surgical intervention [16]. Current treatments are limited but tend to be more effective in earlier stages [14,16].

Clinical suspicion is key in the early identification of PAS. Past medical history and the presence or lack of risk factors in more common diseases are important when investigating non-specific symptoms. Many of the related case reports in the literature note a lack of smoking or tobacco use, which is common in other lung cancer/pathology workups [17]. By doing so, healthcare providers are better able to question the working diagnosis when it does not fit well with the clinical history. In the aforementioned case, the patient did not have any prior cardiovascular disease or traditional risk factors such as smoking, obesity, or atherosclerosis. His acute symptoms should have warranted greater scrutiny in the context of overall health such that new atrial fibrillation would likely not be the primary cause of bilateral saddle pulmonary embolisms. Physicians must remain vigilant and detail-oriented when assessing patients with cardiovascular symptoms such as the ones described in this case report [18-20]. Further studies are needed to better screen and understand the disease progression for PAS. 

## Conclusions

In summary, primary pulmonary angiosarcoma is an exceedingly rare malignant vascular tumor with a nonspecific yet aggressive clinical course. As it stands, the time between the onset of symptoms and definitive diagnosis ranges from two to six months. Treatment tends to be more effective in earlier stages of primary PAS. It is highly suggested that screening and detection criteria allowing for earlier treatment of PAS be developed. 
